# Bacteriophage RNA polymerases: catalysts for mRNA vaccines and therapeutics

**DOI:** 10.3389/fmolb.2024.1504876

**Published:** 2024-11-21

**Authors:** Adithya Nair, Zoltán Kis

**Affiliations:** ^1^ School of Chemical, Materials and Biological Engineering, University of Sheffield, Sheffield, United Kingdom; ^2^ Department of Chemical Engineering, Imperial College London, London, United Kingdom

**Keywords:** bacteriophage RNA polymerase, T7 RNA polymerase, T7 RNA polymerase mutants, RNA polymerase engineering, *in vitro* transcription, mRNA vaccines and therapeutics, mRNA manufacturing, quality by design

## Abstract

Decades of research on bacteriophage-derived RNA polymerases (RNAPs) were vital for synthesizing mRNA using the *in vitro* transcription (IVT) reaction for vaccines during the COVID-19 pandemic. The future success of mRNA-based products relies on the efficiency of its manufacturing process. mRNA manufacturing is a platform technology that complements the quality by design (QbD) paradigm. We applied the QbD framework in combination with key mechanistic insights on RNAP to assess the impact of IVT-associated critical process parameters (CPPs) and critical material attributes (CMAs) on the critical quality attributes (CQAs) of the mRNA drug substance and on manufacturing key performance indicators (KPIs). We also summarize the structure-function relationship of T7 RNAP and its engineered mutants aimed at enhancing the critical production of low-immunogenic mRNA therapeutics. Alternatives to the current set of standard RNAPs in large-scale IVTs are also discussed based on a phylogenetic background. Finally, the review dives into the economic implications of improving mRNA manufacturing based on the main enzyme, T7 RNAP, used to synthesize the mRNA drug substance. The review concludes by mapping the relationship between various CMAs and CPPs with different phases of the IVT reaction from a QbD perspective.

## Graphical Abstract

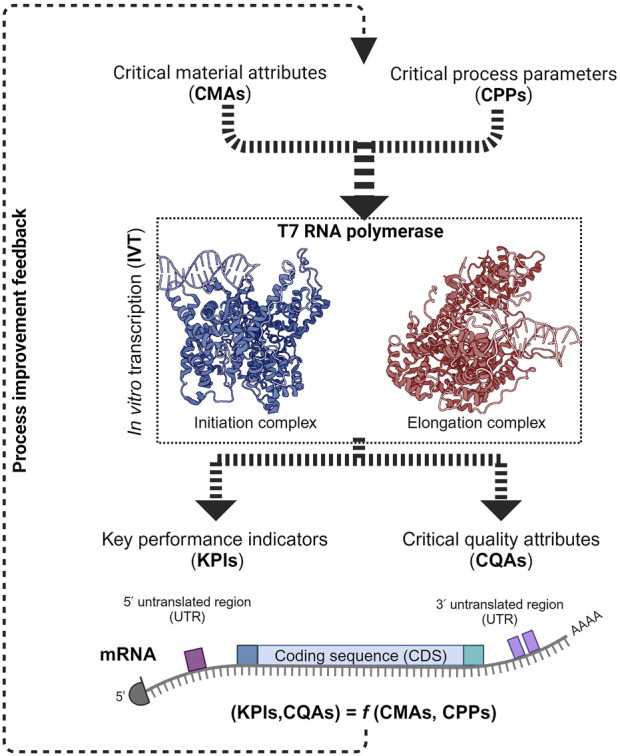

## 1 Introduction

Bacteriophage-derived DNA-dependent RNA polymerases (RNAPs) have been instrumental in cell-free *in vitro* synthesis of RNA. These polymerases play a central role in the *in vitro* transcription reaction (IVT), enabling the production of RNA vaccines and therapeutics from nanograms to the kilogram scale (Krieg and Melton, 1984; Milligan et al., 1987; Yisraeli and Melton, 1989; Dias et al., 2018; Skok et al., 2022a). The transition of phage polymerases from an enzyme for producing small-scale RNA for research purposes to a significant component in the large-scale manufacturing of mRNA vaccines and therapeutics occurred during the COVID-19 pandemic (Kis et al., 2020b; Kis et al., 2020a; Szabó et al., 2022). The synthesis of all regulatory-approved mRNA vaccines utilized the IVT reaction that used the bacteriophage T7-derived RNA polymerase. This transformative mRNA platform technology is advancing the development of a rapidly growing number (already in hundreds) of vaccines and therapeutics against a wide range of diseases, including infectious diseases, cancers, immune disorders, rare diseases, cardiovascular diseases and much more (Kumar et al., 2022; Qin et al., 2022; Whitley et al., 2022). Apart from the conventional type of non-replicating mRNA (used in both the approved SARS-CoV2 vaccines), there is significant interest in developing vaccines and therapeutics based on self-amplifying mRNA (saRNA) and circular mRNA (circRNA) (Bloom et al., 2020; Pisignano et al., 2023; Qi et al., 2023; Jones et al., 2024). Next-generation products based on saRNA and circRNA promise lower dosage, better stability, and longer duration *in vivo* expression. saRNA codes for a viral RNA-dependent RNA polymerase (usually derived from alphaviruses) along with the gene of interest; this coded polymerase is responsible for the *in vivo* amplification of the drug substance (Comes et al., 2023). circRNA forms covalently closed loop structures compared to linear RNA, which are resistant to exoribonuclease digestion and provide better stability (Greene et al., 2017; Bai et al., 2023). Regardless of the type of mRNA, IVT is the choice of synthesis, and bacteriophage-derived RNA polymerases remain the enzymes used for this polymerization reaction.

### 1.1 Discovery and early application

**FIGURE 1:**
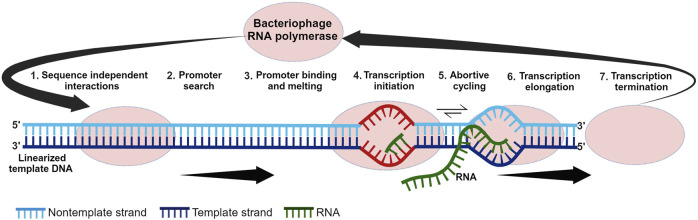
Different phases within the *in vitro* transcription reaction. In step one, the polymerase scans the template DNA for the promoter sequence. Once the promoter is found, it is melted in step three. Transcription is initiated in step four and this is followed by abortive cycling in step five. Promoter escape and transition to elongation occur in step six which is followed by termination in step seven.

The history of the IVT reaction that enables large-scale mRNA synthesis is intertwined with the discovery and characterization of RNAPs. The discovery of mRNA as the intermediary between DNA and protein in the late 1950s to early 1960s prompted the search for the enzyme responsible for mRNA synthesis. This, in turn, led to the discovery of mammalian/bacterial RNAPs in the 1960s and bacteriophage RNAPs later in the 1970s (Weiss and Gladstone, 1959; Hurwitz et al., 1960; Stevens, 1960; Chamberlin et al., 1970; Hurwitz, 2005). The single-subunit RNAPs (ss-RNAPs) from bacteriophages T7, T3, and SP6 were among the first to be discovered, isolated, characterized and synthesized (Chamberlin et al., 1970; Gelfand and Hayashi, 1970; Dunn et al., 1971; Chakraborty et al., 1973; Niles et al., 1974; Butler and Chamberlin, 1982; Kassavetis et al., 1982; Davanloo et al., 1984; Morris et al., 1986; Kotani et al., 1987). RNAPs from T-odd phages (T7 and T3) were the first to be extensively investigated due to research on phage-infected bacteria and their associated gene expression (Summers and Siegel, 1970; Lillehaug et al., 1973; Dunn et al., 1977). These phage polymerases, their corresponding interactions with their specific promoters, and the IVT reaction parameters were studied throughout the 1970s (Dunn et al., 1971; Chamberlin and Ring, 1973a; Bautz et al., 1974; Oakley et al., 1975; Oakley and Coleman, 1977). The extensive research on these phage RNAPs led to the development of an efficient expression system (Davanloo et al., 1984; Moffatt et al., 1984; Tabor and Richardson, 1985; Studier and Moffatt, 1986) and subsequent large-scale *in vitro* mRNA synthesis. Different phases within the IVT reaction are shown in Figure 1.

**FIGURE 2:**
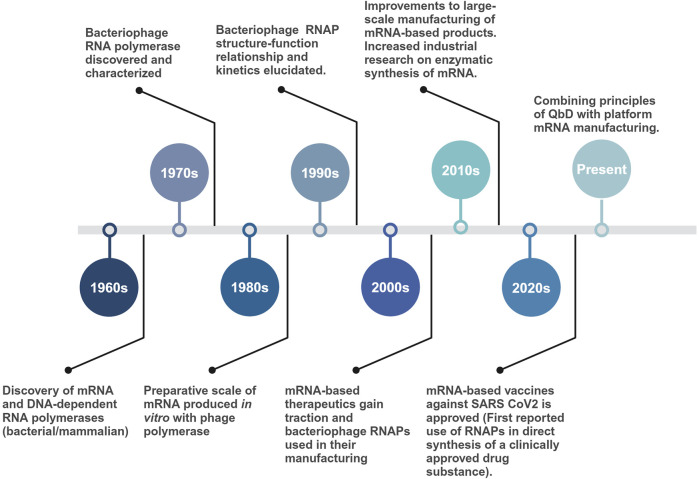
Timeline of major events starting with the discovery of bacteriophage-derived RNAPs to their use in large-scale production of mRNA-based vaccine drug substance. More than five decades of fundamental research on single-subunit RNAPs enabled the rapid manufacturing of mRNA-based vaccines during the COVID-19 pandemic.

Early sequence elucidation of the T7 RNAP, its promoters, and the development of T7-based expression systems made it the gold standard for the current *in vitro* industrial scale production of mRNA and *in vivo* gene expression systems. Even after sharing similarities with the T3 RNAP (83% amino acid sequence similarity), early adoption, high promoter specificity and processivity led to T7 being preferred over other homologous phage polymerases (T3 and SP6) (Eun, 1996). Moreover, for *in vitro* applications with high ribonucleotide (rNTP) concentrations (>20 mM), T7 has proven much more effective than SP6 RNAP (CustomBiotech, 2023). Surprisingly, it was an SP6 RNAP-based IVT (Krieg and Melton, 1984), credited with being first used for synthesizing large quantities of eukaryotic mRNA. Structural and phylogenetic analysis have shown the extensively used T7, T3 and SP6 RNAPs to be related to each other along with other bacteriophage and mitochondrial RNAPs (nuclear gene-encoded and linear mitochondrial plasmid-encoded RNAPs) (Joho et al., 1990; Klement et al., 1990; Jorgensen et al., 1991; McAllister and Raskin, 1993; Cermakian et al., 1997). Similar to the RNAPs, their associated promoters have conserved sequences, suggesting an evolutionary relationship. A timeline of significant events, from the discovery of the first bacteriophage-derived RNAPs to their current large-scale application, is given in Figure 2.

### 1.2 Single-subunit vs. multi-subunit RNAPs

ss-RNAPs are recognized for their structural simplicity, high promoter specificity, and processivity; these properties have made the bacteriophage RNAPs the ideal choice for various research and manufacturing-related applications. Multi-subunit RNAPs (ms-RNAPs), found in eukaryotes, bacteria, archaea, and some viruses, in contrast, are structurally complex (consists of catalytic subunit aided by several accessory subunits) and have lower processivity compared to ss-RNAPs (Chamberlin and Ryan, 1982; Eun, 1996; Sousa and Mukherjee, 2003). Additionally, these ms-RNAPs require specific transcription factors for their function. *Escherichia coli* RNAP (a well-characterized ms-RNAP) consists of 5 subunits (α_2_ ββ′ω) and additional transcription factors (σ), and this complex is around four times bigger than the common ss-RNAPs (T7, T3 and SP6). The multi-subunit bacterial RNAPs are less complex than their eukaryotic counterparts; unlike bacterial RNAPs, there is a broader diversity within the eukaryotic RNAPs used for the synthesis of different types of RNAs (Chamberlin, 1974; Helmann and Chamberlin, 1988; Saltzman and Weinmann, 1989; Horwitz and Loeb, 1990; Woychik and Young, 1990; Eun, 1996).

The eukaryotic RNAPs tend to have more subunits (10–14 subunits for RNAP II) compared to bacterial RNAPs and are 5–7 times bigger than the previously mentioned phage polymerases. Regarding transcription rate, the single-subunit RNAPs are faster, usually 100–200 nucleotide/second (nt/sec) than the multi-subunit RNAPs (usually around 20–50 nt/s) (Eun, 1996; Sousa and Mukherjee, 2003). Furthermore, the promoters for single-subunit RNAPs also tend to be a single continuous block of sequences, unlike those for multi-subunit RNAPs, which tend to have multiple regulatory elements. A disadvantage of the structural and functional simplicity associated with single-subunit RNAPs is reflected in its lack of proofreading mechanisms, which is not observed among its muti-subunit counterparts (Sydow and Cramer, 2009). An interesting difference between single-subunit and multi-subunit RNAPs pertains to the latter’s sensitivity towards antibiotics such as rifamycins (binds to β subunit, prevents transcription elongation) and fidaxomycins (inhibits transcription initiation), making bacterial RNAPs an ideal drug target. Antibiotics that specifically target eukaryotic RNAPs were also screened based on this logic, anti-cancer drug α-amanitin targets RNAP II by preventing translocation of the enzyme and disrupting nucleotide addition in the elongation phase (Chamberlin and Ring, 1972; Chamberlin and Ring, 1973b; Küpper et al., 1973; Ma et al., 2016; Mosaei and Harbottle, 2019; Kirsch et al., 2022). Sensitivity to elevated concentrations of salts is also another difference between single-subunit and multi-subunit RNAPs; while the activity of the former is inhibited, the latter is stimulated/tolerant in the presence of excess salt concentrations (Chamberlin and Ring, 1973b).

### 1.3 Elucidation of transcription mechanisms

Once the amino acid and nucleotide sequences (Moffatt et al., 1984; Mcgraw et al., 1985; Kotani et al., 1987; Dietz et al., 1990) of bacteriophage-related RNAPs were deciphered, these enzymes were overexpressed (Davanloo et al., 1984; Morris et al., 1986) and used extensively in *in vitro* studies to reveal the various mechanistic properties of the transcription reaction. Early studies gave insights into promoter binding, initial template melting and transcription initiation (Bautz et al., 1974; Oakley et al., 1975; Oakley et al., 1979; Bishayee et al., 1976; Martin and Coleman, 1987; Muller et al., 1988). Reaction kinetics were studied to elucidate binding affinity and subsequent reaction rate with Michaelis constant (*K*_m_) for enzyme-promoter binding in the presence of different ions, the initiating nucleotide and subsequent nucleotides (Martin and Coleman, 1987; Martin et al., 1988; Maslak and Martin, 1994). Structure-function studies revealed the role of various domains of the enzyme and their interactions with i) template DNA (based on DNA footprinting studies), ii) the ribonucleotides, iii) the divalent metal ion co-factor (Mg^2+^), iv) nascent RNA, v) RNA-DNA hybrid (within the transcription bubble), vi) growing single-stranded RNA and vii) terminator sequences (Oakley et al., 1975; Ikeda and Richardson, 1986a; Muller et al., 1988; Muller et al., 1989; Basu et al., 1989; Martin and Coleman, 1989). The difference between the initiation complex (IC) and elongation complex (EC) was elucidated, and the processivity of the RNAP in the elongation phase was determined (Martin et al., 1988; Muller et al., 1988). The structure-function analysis also identified which domains of the enzyme were responsible for the major events in each phase during the transcription reaction (Sousa, 1996; Sousa, 1997; Brieba and Sousa, 2000; Brieba and Sousa, 2001; Yin and Steitz, 2002). Moreover, the stoichiometry of the IVT reaction was improved based on the mechanistic and structure-function studies (Maslak and Martin, 1994). These early works also revealed the drawbacks of phage RNAP-assisted IVT reactions. Several product-related impurities, including but not limited to short abortive transcripts, “run-on”/“read-through” transcripts, and double-stranded RNA (dsRNA), were discovered (Milligan et al., 1987; Krupp, 1988; Triana-Alonso et al., 1995). The exact mechanism of generating some of these byproducts (abortive transcripts, dsRNA) has been deciphered, but ambiguity remains for impurities such as run-on transcripts.

### 1.4 T7 RNAP (the mRNA vaccine production gold standard)

**FIGURE 3:**
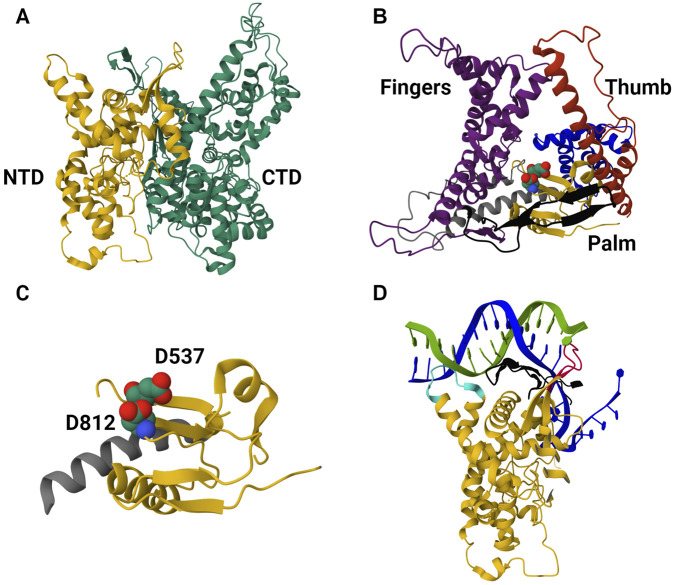
The 3D protein structure of the T7 RNAP in the initiation complex. **(A)** Structure of T7 RNAP in the initiation complex (IC) derived from PDB-1QLN. It is divided into the N-terminal domain (NTD, colored in yellow) and the C-terminal domain (CTD, colored in green). NTD amino acid residues range from 1 to 312, while CTD ranges from 313 to 883. **(B)** Structure of the CTD. The CTD is the catalytic domain (structured like a cupped right hand) of the enzyme with three subdomains, namely, i) “fingers” (colored purple, residues 541 to 737 and 771–778), ii) “palm” (colored yellow, residues 411 to 448, 532 to 540 and 788–838) and iii) “thumb” (colored red, residues 330–410). **(C)** The palm subdomain of the CTD. Residues D537 and D812 in space-filling representation (close to the C-terminal loop). These residues coordinate with the co-factor (Mg^2+^) and facilitate phosphodiester bond formation between ribonucleotides. **(D)** The NTD binding to the promoter region of the template DNA. The NTD and promoter recognition loop bind to the promoter region of the double-stranded template DNA and open it up to start transcription initiation. Positively charged residues from 91 to 103 within the NTD (colored cyan) interact with the minor groove of the promoter (from −17 to −13 bp). An intercalating loop (colored red) formed by residues 232 to 242 opens the DNA duplex and stabilizes the upstream edge of the bubble. The promoter recognition loop formed by residues 739 to 770 (colored black, antiparallel *β* ribbon) interacts with the major groove in a sequence-specific manner.

T7 bacteriophage-derived RNAP is most often used for *in vitro* synthesis of mRNA-based products; this enzyme has practically served as a model for understanding single-subunit RNAPs as well as the transcription reaction in general (McAllister, 1993; Eun, 1996; Yin and Steitz, 2002; Sousa, 2013). T7 RNAP has been successful in its industrial applications, and extensive knowledge of its structure and structure-function relationship has made it the ideal candidate for IVT optimization. The enzyme characterization began shortly after its discovery in the 1970s (Chamberlin and Ring, 1973a); studies on T7 RNAP inhibition and its interactions with the T7 promoter were among the initial findings (Chamberlin and Ring, 1973b; Chamberlin and Ryan, 1982). Elucidation of the T7 RNAP transcription mechanism began with studies on promoter binding and transcription initiation; the interactions between T7 RNAP and its corresponding Class II and III promoters were also studied (this helped optimize IVT parameters such as ionic strength and reaction temperature) (Mcallister and Carter, 1980; Carter and McAllister, 1981). The structural simplicity of the T7 RNAP was also responsible for its extensive use in studying the transition of RNAPs from initiation to elongation complex (Jia and Patel, 1997; Yin and Steitz, 2002; Skinner et al., 2004; Tang et al., 2009; Koh et al., 2018). Sequence-dependent and independent transcription termination mechanisms were also elucidated to give a better mechanistic understanding of transcription (Macdonald et al., 1994; Lyakhov et al., 1998). Decades of structural studies from the late 1980s revealed the 3D structure and the structure-function properties of T7 RNAP. The enzyme is broadly split into its amino-terminal domain (N-terminal domain, NTD) and carboxyl-terminal domain (C-terminal domain, CTD); the latter is further divided into “fingers,” “palm,” and “thumb” subdomains (Sousa, 1996; Sousa, 1997; Cheetham et al., 1999; Yin and Steitz, 2002; Yin and Steitz, 2004). CTD is the main polymerase domain of the enzyme, and the function of each subdomain has been elucidated with structure-function studies. Moreover, these studies have also been used for phylogenetic analysis to reveal homology with closely related RNAPs and structural similarities with distantly related RNAPs, pointing to the convergent evolution of RNAPs. The NTD is among the “accessory” modules along with the promoter recognition loop (inserted within the CTD), the C-terminal loop, and the 4-helix bundle (Sousa and Mukherjee, 2003; Sousa, 2013). T7 RNAPs were also subjected to extensive kinetic studies; although these were initially done to understand the different mechanisms involved in the transcription reaction, the insights derived from these studies lay the foundation for large-scale synthesis of *in vitro* transcribed mRNA (Young et al., 1997; Rosa et al., 2022; Samnuan et al., 2022). Various computation models simulating the IVT reaction resulted from these extensive studies on T7 RNAP-enabled IVT reaction (Akama et al., 2012; van de Berg et al., 2021; Stover et al., 2024). T7 RNAP structure in the IC is shown in Figure 3.

### 1.5 Drawbacks associated with phage RNAPs

As mentioned, the product-related impurities generated during the IVT reaction can be traced back to specific RNAP mechanisms. Once mRNA-based vaccines and therapeutics gained traction, these impurities and their impact on patient safety were scrutinized (Mu et al., 2018; Lenk et al., 2024). Even though these impurities can be removed with various downstream purification techniques, the associated costs pose a significant hurdle (Kis et al., 2020a). RNAPs during IVT produce impurities such as dsRNA, abortive transcripts (product of abortive cycling), and run-on transcripts. Additionally, the mRNA-based products may require the incorporation of modified nucleotides into the transcript; this could lower the activity of wild-type RNAPs used to synthesize the drug substance (Nelson et al., 2020; Chen et al., 2022). The single-subunit RNAPs also lack the capabilities of 5′ modifications (5′ cap); this is offset by either post-transcriptional capping or co-transcriptional capping using cap analogs such as ARCA (dinucleotide) or CleanCap® (GAG trinucleotides) (Hogrefe et al., 2017; Roy and Ong, 2021). Post-transcriptional enzymatic capping is an effective method but requires additional purification after the initial synthesis and an additional enzymatic reaction. There is ongoing work to optimize a “co-transcriptional” one-pot IVT and enzymatic capping reaction (Nwokeoji et al., 2023), including the use of fusion proteins between the T7 RNAP and capping enzymes. Co-transcriptional analog-based capping is hindered by the lower capping efficiency seen in the case of ARCA or cost burdens due to proprietary CleanCap® technology. Regardless, a one-step synthesis stage can be more attractive in terms of process productivity and manufacturing costs (Wang et al., 2017; Chan et al., 2023). The limitations of bacteriophage-derived RNAPs in the context of product-related impurities have led to the development of solutions that include mutant T7 RNAPs and RNAPs derived from less commonly used bacteriophages. A summary of such mutants and alternative RNAPs is given in Tables 1, 2, respectively. These solutions claim to reduce the generation of product-related impurities and improve the quality of the drug substance at the synthesis stage. However, thorough analysis and validation is required before they can replace the current standard RNAPs.

**TABLE 1:** List of T7 RNAP mutants and their improved functions.

Source	Type of modification	Residue location	Domain position	Application
Ikeda (1995)	Substitution	E222K	NTD	Modified promoter recognition
Sousa and Jendrisak (2000)	Substitution	Y639F	Fingers	2′-fluoro-nucleoside incorporation
Sugiyama et al. (2009)	Substitution	S430P, N433T, S633P, F849I, F880Y	Fingers, Palm	Thermostability
Padilla and Sousa (2002)	Substitution	Y639F, H784A	Fingers, Palm	2′-fluoro-nucleoside incorporation
Chelliserrykattil and Ellington (2004)	Substitution	E593G, Y639V, V685A, H784G	Fingers, Palm	2′-O-methyl-nucleoside incorporation
Chelliserrykattil and Ellington (2004)	Substitution	G542V, H772R, H784S	Fingers, Palm	2′-fluoro-nucleoside incorporation
Guilleres et al. (2005), Guilleres et al. (2004)	Substitution	P266L	NTD	Reduced 8 nt abortive transcription
Siegmund et al. (2012)	Substitution	I119V, G225S, K333N, D366N, F400L, Y639V, S661G, H784G, F880Y	NTD, Fingers, Palm, Thumb	2-Se-methyl-UTP and 2′-O-methyl-UTP incorporation
Brakmann and Ibach (2015)	Substitution	R425C	Palm	2′-O-methyl-nucleoside incorporation
Meyer et al. (2015)	Substitution	S430P, N433T, E593G, S633P, Y639V, V685A, H784G, F849I, F880Y	Fingers, Palm	2′-modified-nucleoside incorporation, improved activity
Ellington and Meyer (2018)	Substitution	S430P, N433T, G542V, S633P, H772R, H784S, F849I, F880Y	Fingers, Palm	2′-modified-nucleoside incorporation
Sobek et al. (2016)	Substitution	V426L, A702V, V795I	Fingers, Palm	Thermostability
Greif et al. (2017)	Substitution	C723S	Fingers	Stability (reduced homodimers)
Martin and Ramirez-Tapia (2015)	Insertion	G-ins-E252, G-ins-G259, G-ins-P266	NTD	Reduced abortive transcripts
Ong et al. (2019)	Substitution	T75Q, A83K, I109L, H205S, K206P, I281P, A327P, T375K, D388E, L446F, C510Q, L534V, V567P, G618Q, K642R, M832F, D834E, S856T, A863P, A866K	NTD, Fingers, Palm, Thumb	Thermostability
Jain (2021)	Substitution	I320L, I396L, F546W, S684A, G788A	Fingers, Palm, Thumb	Thermostability, reduced dsRNA
Miller et al. (2022)	Substitution	P664W	Fingers	Cap analog incorporation
Thompson et al. (2022)	Substitution	P266L, K378R, S430P, N433T, S633P, Y639L, H784A, F849I, F880Y	NTD, Fingers, Palm, Thumb	2′-modified-nucleoside incorporation, thermostability
Oe et al. (2013)	Substitution	K179, V685A, Q768	NTD, Fingers	Thermostability
Rabideau et al. (2019)	Insertion, Substitution	S43A, G47A, R257A G-ins-844	NTD, C-terminal	Reduced dsRNA
Wu et al. (2021)	Substitution	S43Y	NTD	Reduced dsRNA

**TABLE 2:** List of commonly used and alternative bacteriophage RNAPs.

Source	Name	Promoter	Total number of amino acid residues	Genbank accession number
Xu et al. (2020)	*Yersinia* phage phiR8-01	TCGACCCTATTAAAC	810	CCI88411.2
	*Aeromonas* phage phiAS7	TTGATTCGGTACGCCTAA	816	YP_007007815.1
	*Caulobacter* phage Percy	ACATTCTCGCTACACCAA	805	YP_009225265.1
	Burkholderia phage Bp AMP4	TTTCGGTCGCCTTACCGACAC	831	CDL65264.1
	*Pseudomonas* phage Andromeda	CCACTATAGCAACA	803	YP_009279548.1
	*Proteus* phage vB_PmiP Pm5460	TAATTAGAGACCACTATA	875	YP_009209198.1
	Delftia phage IME-DE1	GTTAGCCCACACCATT	859	YP_009191807.1
	*Vibrio* phage N4	AATTAACCCACACTATA	883	YP_003347903.1
	*Morganella* phage vB_MmoP MP2	ACATTTGTGGCACTATA	883	YP_009291533.1
	Xanthomonas phage f30-Xaj	TTGGTACACCTATA	836	YP_009276314.1
	*Escherichia* phage T7	TTAATACGACTCACTATA	883	QZB83517.1
	Pantoea phage LIMElight	TGACGTTATAGAGAGACAAC	818	YP_007002889.1
	*Salmonella* virus SP6	ATTTAGGTGACACTATA	874	NP_853568.1
	*Escherichia* phage ECBP5	TAGGCACTACAATA	877	YP_009146377.1
	Kluyvera phage Kvpl	AATACGACTCACTATT	882	YP_002308386.1
	*Klebsiella* phage KP32	ATTAGGGCACACTATAG	906	YP_003347522.1
	Stenotrophomonas phage IME15	TTAATACGACTCACTATAGGGAGA	883	YP_006990207.1
	Enterobacteria phage T3	AATTAACCCTCACTAAAGGG	884	NP_523301.1
Lu et al. (2019)	*Klebsiella* phage KP34	TAATGTTACAGGAGTA	822	YP_003347629.1
Zhu et al. (2014)	Cyanophage Syn5	ATTGGGCACCCGTAA	779	YP_001285424.1
Xia et al. (2022)	Psychrophilic phage VSW-3	TTAATTGGGCCACCTATA	798	YP_009596173.1
Streit et al. (2023)	Phage_EMG_100,139,454	TCAGAAGTCACACTATAA	816	UVM79537.1

### 1.6 Mutant T7 RNAPs

As T7 RNAP was characterized extensively, its structure and structure-related functions were modified to make mutant RNAPs with a reduced product-related impurity footprint. The wild-type T7 RNAP also has limited capabilities in synthesizing transcripts containing modified nucleotides. These substrate modifications could include 2′ modified ribose, base modifications (Ψ Uridine, N1-methyl-Psuedouridine) or 5′ cap analogs (ARCA, GAG) (Siegmund et al., 2012; Meyer et al., 2015; Chen et al., 2022; Miller et al., 2024). Modifications to amino acid sequence in the T7 RNAP finger subdomain, responsible for interactions with the substrate, are widely employed for incorporating substrates other than wild-type ribonucleotides. The palm subdomain is also modified to incorporate alternative substrates. A solution to reduce the synthesis of immunogenic dsRNA is the use of RNAPs at higher reaction temperatures (>45°C) (Wu et al., 2020; Wu et al., 2021; Roy and Robb, 2022). This was achieved with modifications to the amino acid sequence throughout the T7 RNAP; in most cases, the CTD was modified. Apart from thermostability, properties such as structural stability were also considered, such that the enzymes do not form homodimers and reduce the overall enzymatic activity. Modifications to NTD, specifically the linker between NTD and CTD, have been targeted to make mutant T7 RNAPs that generate fewer abortive transcripts by facilitating an easier transition to EC from the IC (Wu et al., 2021; Dousis et al., 2023; Rabideau et al., 2019). The C-helix within the NTD is also targeted to achieve the same IC to EC transition. Additions to the end of CTD were previously observed to be detrimental to the enzyme’s function (Mookhtiar et al., 1991; Gardner et al., 1997), but newer studies have found CTD insertional mutants to be functional and effective in the reduction of run-on transcripts (Dousis et al., 2023).

### 1.7 Alternatives to T7 RNAP

Apart from modifications to the well-characterized bacteriophage RNAPs, other lesser-known phage RNAPs and modified DNA polymerases are also being considered for industrial-scale production of mRNA. These RNAPs are assumed to provide certain advantages over the current industry standards by producing fewer product-related impurities. Most of these belong to the *Autographiviridae* family of viruses (Zhu et al., 2014; Lu et al., 2019; Xu et al., 2020; Xia et al., 2022; Streit et al., 2023; Curry et al., 2024). The search for alternative phage RNAPs other than T7, T3, SP6 and K11 for IVT is reflected in the increasing number of research articles and patent applications (summarized in Table 2); although these new RNAPs are touted as a solution to the current standards, their extensive characterization and effectiveness is yet to be established. Similar to the development of mutant T7 RNAPs, mutants of these new alternative RNAPs are also being explored for improved activity and wider substrate type utilization (Zhu et al., 2015). Mutated DNAPs from extremophiles (e.g., *Thermococus gorgonarius*) are also reported to be helpful in the synthesis of mRNA using IVT, especially at elevated incubation temperatures (>45°C) (Wang et al., 2017).

### 1.8 IVT improvement strategies

Immobilized RNAP and template DNA in an IVT reaction are also explored to reduce the synthesis of product-related impurities. The proximity of RNAP and template DNA, along with IVT process parameters such as high ionic strength, is used to reduce the rebinding of RNAP onto the synthesized mRNA, thus mitigating the RNA-dependent RNA polymerase activity (Cavac et al., 2021; MalagodaPathiranage et al., 2023). Immobilized RNAPs, commonly done with streptavidin, have been previously employed for single-molecule analysis to elucidate the transcription kinetics (Skinner et al., 2004). Apart from the benefits of reducing product-related impurities, immobilization also helps reduce manufacturing costs by potentially aiding the reuse of the RNAP (Malag et al., 2024). Raw materials for mRNA synthesis are the highest manufacturing cost contributors; a significant fraction comes from the RNAP costs. The benefits of enzyme immobilization become much more apparent while transitioning from a batch to a flow-based continuous manufacturing mode (Wochner et al., 2015; Kis et al., 2020b).

### 1.9 Quality by design to improve IVT

As phage RNAP is a central element of the IVT reaction, it directly affects the mRNA’s critical quality attributes (CQAs). Product-related impurities synthesized as byproducts, along with the intended transcripts, affect the purity of the drug substance (DS) (Lenk et al., 2024; Popova et al., 2024). The IVT process parameters can also influence the fidelity of the RNAP and may cause errors in the transcripts that would cause a decrease in the effectivity of the DS *in vivo* or even safety issues. The integrity (intactness of the transcript) is also affected by the IVT process parameters due to their effect on the RNAP and the transcription complex. Therefore, the critical process parameters (CPPs) and critical material attributes (CMAs) that affect the RNAP activity must be identified, mapped and optimized to synthesize the intended transcript with the target CQAs under efficient manufacturing conditions. Understanding the effect of CPPs and CMAs on the RNAP activity at different phases of transcription reaction becomes essential in implementing the quality by design (QbD) approach in manufacturing mRNA-based products (Daniel et al., 2022; Nair et al., 2024). QbD implementation also has the added advantage of efficiently using prior knowledge and assisting with subsequent approvals as long as the reported design space is maintained. mRNA manufacturing also has the unique advantage of using similar unit operations for the production of multiple products by only changing the transcript encoding template DNA; this property makes it a platform technology and the manufacturing knowledge from one product is easily transferable to another. Pharmaceutical manufacturing is increasingly moving towards the QbD paradigm and mRNA manufacturing, with its “platformability”, complements this approach.

## 2 RNAP mechanisms in *in vitro* transcription

The discovery of single-subunit RNAPs from bacteriophages significantly helped elucidate the transcription reaction. Steps like template scanning, promoter binding, transcription initiation, abortive cycling, processive elongation and termination have been studied in detail and the reaction parameters that influence these steps have been determined. The following sections will look at these distinct phases in the context of *in vitro* transcription and their corresponding RNAP mechanisms.

### 2.1 Promoter search

**FIGURE 4:**
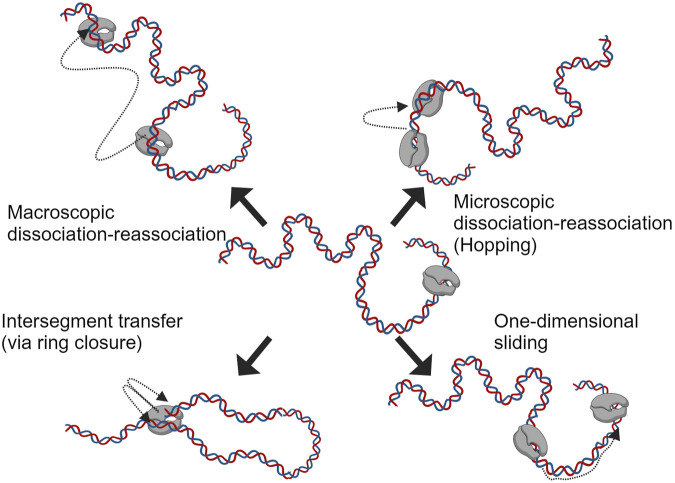
Different mechanisms speculated in aiding facilitated diffusion during promoter search by RNAP on template DNA (adapted from Berg et al., 1981).

Before the RNAP binds to the promoter region and initiates the transcription, it searches/scans for it on the template DNA with intermittent weak interactions in a sequence-independent manner (Skinner et al., 2004; Kim and Larson, 2007). This phenomenon is often explained using diffusion processes (driven by thermal fluctuations) and has been studied extensively for single and multi-subunit RNAPs (Park et al., 1982; Guthold et al., 1999). Studies on protein-nucleic acid interactions have suggested four mechanisms (Berg et al., 1981) for the translocation of polymerases on nucleic acids; these are macroscopic dissociation-reassociation, microscopic dissociation-reassociation (hopping), intersegment transfer and sliding (one-dimensional diffusion); these mechanisms are depicted in Figure 4. The sequence-independent interaction of RNAPs with DNA has been studied further with single-molecule assays in combination with fluorescence microscopy, total internal reflection fluorescence (TIRF) with optical trapping and atomic force microscopy (AFM) (Guthold et al., 1999; Harada et al., 1999; Lee and Myong, 2021). The results from the assays mentioned above suggest a linear motion of RNAP on the template during promoter search; a more complex grove-tracking motion was also suggested, considering the double-helical structure of the DNA (Harada et al., 2001). The outcome of all these studies has led to the consensus that facilitated diffusion helps with promoter search (Bai et al., 2006). For the T7 RNAP, the NTD was observed to be responsible for nonspecific polynucleotide interactions, and the nicking/extensive proteolysis of this domain shows reduced interaction of the RNAP with nonspecific DNA (Muller et al., 1988). The property of RNAPs to interact with nonspecific DNA segments as in the case of searching for the promoter site, is sometimes adapted to sequester/quench RNAP activity (useful for single-round transcription studies or minimizing RNA-dependent RNA polymerase activity by competing with synthesized transcripts) (Chamberlin and Ring, 1973a; Chamberlin, 1974; Gholamalipour et al., 2019). In theory, CMAs such as template DNA length, could be optimized to reduce the nonspecific DNA interactions that the RNAP encounters in an IVT reaction. Linearized plasmid DNA amplified using bacterial fermentation contains a considerable amount of sequences (e.g., antibiotic resistance genes that act as selectable markers, origins of replication, multiple cloning sites, copy number control elements, etc.) not relevant for the final product. These could be reduced by using cell-free enzymatically prepared templates such as PCR products or proprietary templates based on dbDNA™, oeDNA™ or opDNA™ technology (Adie et al., 2022; Barreira et al., 2022; Cameron, 2024; Dhir et al., 2024).

### 2.2 Promoter binding and melting

**FIGURE 5:**
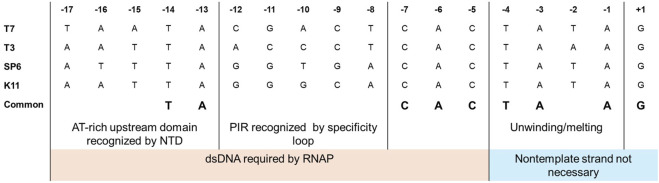
Comparison of promoters for commonly used bacteriophage RNAPs. A two-domain structure can be observed for the promoters, with upstream elements from −17 to −5 involved in recognition/binding and initiation elements from −4 to +3 (adapted from Imburgio et al., 2000; Sousa and Mukherjee, 2003).

RNAP-promoter binding follows a two-step mechanism, and initial interactions are like any other weak nonspecific RNAP-template DNA interactions. Once the RNAP recognizes the promoter site, it binds to it and forms the closed initiation complex (Újvári and Martin, 1996; Bandwar and Patel, 2002; Tang and Patel, 2006a). It has been observed with footprinting assays that RNAPs recognize one face of the DNA duplex (Oakley et al., 1979; Strothkamp et al., 1980). The NTD and the promoter recognition loop recognize the promoter region in bacteriophage-derived RNAPs. For the T7 RNAP, amino acid residues 93–101 in the NTD bind to the promoter’s upstream −13 to −17 AT-rich region (Sousa and Mukherjee, 2003). The promoter recognition loop (residues from 739–770) in the T7 RNAP is an insertion within the polymerase domain that recognizes the −12 to −8 promoter identity region (PIR) within the T7 promoter, and it is this interaction that confers specificity between the homologous T3 and T7 RNAPs with their corresponding promoters (Bailey et al., 1983; Li et al., 1996; Cheetham and Steitz, 1999). The nucleotide at positions −11, −10 and −12 were determined to be the main specificity determinants between T3 and T7, while it was −9 and −8 for SP6 and T7 (Klement et al., 1990; Jorgensen et al., 1991). Mutations in the specificity loop and changes to the sequence within the PIR have been done to confirm the promoter recognition capabilities of the standard bacteriophage-derived RNAPs (T7, T3 and SP6) (Rong et al., 1998a). The entire consensus promoter can be divided into two regions: the upstream recognition/binding element (−17 to −5) and the downstream initiation element with melting/unwinding region from −4 to −1 (usually AT-rich, a TATA box in case of class III T7 promoter) and the initial transcription region from +1 to +4 (Carter and McAllister, 1981; Chapman and Burgess, 1987; Imburgio et al., 2000). Extensive studies on the promoter sequences have revealed the relevance of each domain within the consensus promoter and its interactions with the corresponding bacteriophage RNAPs (Chapman and Burgess, 1987; Li et al., 1996). The elements upstream of −5 are required in the double-stranded form, while the nontemplate strand from the −4 position can be removed without hindering the transcription reaction (Maslak and Martin, 1993). A comparison of promoters from commonly used bacteriophage RNAPs is given in Figure 5. Studies with partially single-stranded/nicked promoters (downstream of −5) have shown improved binding kinetics without hindering promoter recognition. Substitutions in the recognition regions have shown greater effects on RNAP binding (*K*_m_) with little effects on the catalytic activity (*k*_*cat*_). In contrast, substitutions in the melting and initiation regions have shown a greater impact on initiation than on binding (Ikeda and Richardson, 1986a; Újvári and Martin, 1997; Weston et al., 1997; Imburgio et al., 2000). It should be noted that for the T7 RNAP, the promoter recognition is very specific but surprisingly weak (Villemain et al., 1997). The T7 class II promoters are weaker than class III promoters and show higher sensitivity to IVT parameters such as temperature and ionic strength (Mcallister and Carter, 1980).

The impact of different types of anions and cations, along with additional reagents like polyamines (spermidine) have suggested optimum levels of these components to improve the key performance indicators (KPIs) for the IVT reaction and CQAs of the transcribed mRNA (Maslak and Martin, 1994). The effect of acetate, chloride and glutamate ions along with their counter ions (Na^+^ or K^+^) on the binding kinetics were explored and have led to unprecedented improvements in the IVT reactions (Boman et al., 2024). It is theorized that these ions compete with the binding of the phosphate backbone of the template DNA, as most of the initial interactions are electrostatic in nature. Acetate and glutamate anions interact less with the RNAP binding site, as evidenced by the higher tolerance of these anions by the RNAP. Moreover, as binding is a diffusion-led phenomenon, the reaction temperature acts as a CPP for this step. Optimization of this CPP based on the temperature tolerance of the RNAP is also reported; 37°C is considered optimal for standard wild-type RNAPs (Maslak and Martin, 1994).

### 2.3 Transcription initiation

Following the binding process, the RNAP melts the promoter to form the open initiation complex (this isomerization is achieved by conformational changes in the RNAP and the template DNA). Thereby, in the case of T7 RNAP-promoter complex, the template is melted from the −4 to +3 position with respect to the transcription initiation site (Cheetham et al., 1999; Újvári and Martin, 2000; Tang and Patel, 2006b; Tang and Patel, 2006a). Rapid promoter opening is synchronized with promoter binding, and it is assumed that the closed and open complexes remain in a state of equilibrium, with closed complexes favored until the initiating nucleotide stabilizes the open complex. The conformational changes associated with transcription initiation include an approximately 90° bend of the downstream template DNA around the −1 site. After the initiating NTP along with the +2 NTP stabilizes the open complex and the first phosphodiester bond is formed to produce a ribonucleotide dimer, this process is repeated until a trimer is formed without any changes in the initial transcription bubble (−4 to +3) (Kuzmine and Martin, 2001; Stano et al., 2002; Tang and Patel, 2006a; Tang and Patel, 2006b; Tang et al., 2009). The rate constant of making a the 2 nt dimer is approximately 2 s^-1^, this decreases as the length of the transcript increases and gets maintained at 0.8 s^-1^ in the elongation phase (Tang et al., 2009). In theory, using dinucleotide or trinucleotide cap analogs skips the first phosphodiester catalysis and may provide better open complex stabilization and initiation of transcription. Most of the energy required for melting/opening of the promoter comes from the binding of the RNAP with the upstream duplex element of the promoter (−17 to −5) (Újvári and Martin, 1996; Bandwar and Patel, 2002; Bandwar et al., 2002). The melting is further facilitated by the interaction of the template strand with the active site cleft and the insertion of the *β*-hairpin formed by the residues 232–243 in the RNAP between the template and nontemplate strands (Cheetham et al., 1999; Stano and Patel, 2002; Sousa, 2013). The upstream edge of the bubble is stabilized by V237 stacking on the promoter’s −5 bp (Brieba and Sousa, 2001; Liu and Martin, 2001).

For T7 RNAP, the initiating nucleotide is mostly GTP, and its binding to the active site is facilitated by the base pairing with the template strand along with Hoogesteen pairing of N-6 and O-6 of the guanine base with either R425 or R632 and interactions with H784 (Kuzmine et al., 2003). It should be noted that the *K*_m_ for the initiating nucleotide is much higher than the rest of the nucleotides and that the second nucleotide is recruited first during synthesis of the first phosphodiester bond (Martin and Coleman, 1989; Jia and Patel, 1997; Stano et al., 2002). The initiating NTP has its triphosphate group intact, while the second NTP recruited to the transcription site loses a pyrophosphate (PPi) group during the bond formation. There are some ambiguities regarding the opening of the promoter being a rate-limiting step; initial experiments with partially single-stranded promoters (no nontemplate strand downstream of −5) and double-stranded promoters did not suggest any drastic difference for the initiation rates and *K*_m_ for binding. However, later studies have proved improved stability and binding while using partially single-stranded promoters (Jia et al., 1996; Újvári and Martin, 1996; Jia and Patel, 1997; Villemain et al., 1997). The inherent instability of the open complex, which is a function of the promoter sequence in the melting and initiation region along with its interaction with the RNAP, acts as a kinetic mechanism for promoter specificity (Villemain et al., 1997). CMAs such as the sequence of template DNA, presence of nontemplate DNA (double-stranded vs. partially single-stranded DNA) at the melting and initiation region, along with CPPs such as concentration of +1 and +2 NTPs or cap analogs (either dinucleotide or trinucleotide) that helps with stabilization of the open complex should be considered for optimization of the IVT reaction. Additionally, metal ions and polyamines such as Mg^2+^ and spermidine and their interaction with the template DNA have also been reported to stabilize the open complex, thereby adding their concentrations to the list of potential CPPs that impact this phase of the IVT reaction.

### 2.4 Abortive cycling

The initial transcription bubble can accommodate up to a trimer of the nascent mRNA before additional conformational changes are required to extend the ribonucleotide polymer. The downstream boundary of the transcription bubble expands downstream from +4 to +8 while still maintaining contact with the promoter (the upstream edge of the bubble remains fixed) (Cheetham and Steitz, 1999; Liu and Martin, 2002; Gong et al., 2004). This initial stage of polymerization, called abortive cycling, is a rate-limiting step before the enzyme transitions to a processive elongation phase. Essentially, the template DNA is “scrunched” within the transcription bubble until the nascent mRNA reaches a minimal length of 8 nt (Cheetham and Steitz, 1999). The promoter release phenomenon as a function of growing nascent RNA (RNA:DNA hybrid) is well documented with exonuclease digestion and fluorescent studies by using promoters with fluorescent base analogs (Liu and Martin, 2002; Gong et al., 2004). The transcription bubble expansion proceeds after each nucleotide incorporation as the RNAP translocates on the template DNA.

The phenomenon of initial transcription proceeding without losing the upstream promoter complex induces template DNA bending and rotation of the NTD of the RNAP. It has been shown that the template DNA is bent almost 90^◦^ in the IC compared to a much-relaxed angle close to 40^◦^ in the EC (Tang et al., 2008). The conformational changes in the RNAP result from the growing RNA:DNA hybrid bumping against the NTD, causing its rotation by 40°, which ends with a large 220° rotation of the same domain during the transition to EC (Yin and Steitz, 2002; Bandwar et al., 2007; Durniak et al., 2008; Tang et al., 2008; Vahia and Martin, 2011). This rotation of the NTD helps with the abrogation of promoter contact and clears the RNAP to a processive elongation phase. During the abortive cycling, a reciprocal pushback by the NTD on the RNA:DNA hybrid causes the release of the nascent mRNA. Multiple rounds of this back and forth between RNAP and the growing RNA:DNA hybrid is necessary before the promoter clearance (Koh et al., 2018). The length of the RNA:DNA hybrid at 8 bp provides a topological lock and stabilizes the transcription bubble, which is the same topological lock that provides stability to the transcription bubble in the EC (Liu and Martin, 2009). RNAP undergoes additional conformational changes, including bending of the thumb subdomain, while the nascent RNA reaches a length of 5–6 nt. Abortive cycling can lead to the RNAP either reinitiating the transcription on the same template (RNAP recycling) or starting it on a new one (RNAP exchange) (Koh et al., 2018). The reinitiations were observed to depend on the RNAP concentration or the initiating nucleotide (GTP) for RNAP exchange and RNAP recycling, respectively. At high GTP concentrations, RNAP recycling was preferred. Abortive cycling ends with a transition to EC following the NTD rearrangement; RNAPs lose the promoter specificity and begin sequence-independent processive elongation. Moreover, the T7 RNAP’s H-subdomain within the NTD is rearranged to form the RNA exit channel (Yin and Steitz, 2002; Tang et al., 2008). The chemical energy from phosphodiester bond formation during the nascent RNA synthesis is converted to mechanical energy during the NTD rotation. It acts as a piston for the RNAP to overcome promoter contact and transition to EC. Abortive cycling is observed in all RNAPs and some have suggested an evolutionary prerogative for its presence. It is assumed that the short nucleotide released during abortive cycling acts as primers for polymerases that do not have *de-novo* synthesis capabilities (DNA polymerases) (Matsumoto, 1994).

As abortive cycling introduces byproducts that impact the quality of the drug substance, CMAs and CPPs that affect this phase need to be optimized. CMAs affecting abortive transcription include a sequence of the initially transcribed RNA (>8 nt). It has been shown that the presence of U in this sequence can lead to higher rates of abortive transcription (based on single nucleotide substitutions) (Imburgio et al., 2000). However, newer data with multiple base substitutions in the initially transcribed sequence (ITS) have highlighted cross-talk between the bases from +4 to +8, and the inclusion of AT-rich regions here has been reported to improve IVT productivity and a reduction in product-related impurities arising from abortive transcription (Conrad et al., 2020; Sari et al., 2024). The structure of the promoter is also reported to be an important influencing factor for abortive cycling, it has been shown that partially single-stranded (duplex promoter and only template strand) or nicked promoters (in the nontemplate strand) reduce the generation of abortive transcripts. It is proposed these modified promoters induce less stress in the transcription bubble by reducing the degree of template DNA scrunching (Gong and Martin, 2006).

CMAs pertaining to the RNAPs have also been extensively studied and used to reduce the abortive cycling phase. Initially, the promoter binding affinity was attributed to be the main factor in preventing the transition to the elongation phase; this was supported by studies based on a T7 RNAP mutant with P266L substitution (Guilleres et al., 2005; Guilleres et al., 2004). Later, it was proved that the binding affinity for the wild-type and the mutant were not significantly different and that the mutant synthesized longer abortive transcripts than the wild-type RNAP. Based on the NTD pushback theory, multiple mutant RNAPs have been engineered to reduce the abortive transcription by easing this phenomenon (Ramírez-Tapia and Martin, 2012; Tang et al., 2014). Substitutions, insertions and deletions in the C-helix of NTD and the linker region between NTD and CTD are reported to reduce the abortive transcripts (Martin and Ramirez-Tapia, 2015). Abortive cycling has been of great interest for IVT optimization studies as it acts as a rate-limiting step before the RNAP transitions to the processive elongation phase, and the byproduct from this mechanism is a product-related impurity that affects the KPI of the IVT reaction and the CQAs of the transcribed drug substance. CMAs, such as template sequences, has been optimized to reduce abortive cycling.

### 2.5 Processive elongation

The transition from the initiation phase through abortive cycling to the processive elongation phase happens after structural changes to both the RNAP and the template DNA. Although the transition to EC proceeds after promoter clearance and the synthesis of >8 nt RNA, the highly processive and stable EC (mature EC) does not form until 12–14 nt RNA is synthesized (Mentesana et al., 2000). Bacteriophage-derived RNAPs catalyze the synthesis of the RNA polymer close to 200 nt/s in this elongation phase (Golomb and Chamberlin, 1974). The transcription bubble stability in the elongation phase is maintained by an almost 8 bp long RNA:DNA hybrid (based on the topological lock) and interactions of the nontemplate strand with the RNAP (Sousa and Mukherjee, 2003; Theis et al., 2004; Liu and Martin, 2009). In one of the main conformational changes to the RNAP in the EC, the specificity loop is displaced from the position in the IC and forms part (lid) of the RNA exit channel. The changes in the NTD are also well characterized; the promoter binding domain (PBD) undergoes a rigid body rotation of 220^◦^ along with changes to the C-helix (transition from loop to helix) and H domain (becomes part of the RNA exit channel) (Yin and Steitz, 2002; Steitz, 2009).

The Brownian ratchet mechanism explains the process of nucleotide addition; the catalysis itself proceeds via a two-metal ion mechanism (divalent metal ions such as Mg^2+^ facilitate this step) (Sosunov et al., 2005; Guo and Sousa, 2006). Apart from the RNAP, the template DNA also undergoes specific changes after transitioning to EC. The downstream DNA is less bent and positioned differently in the EC, for the T7 RNAP, residues K711, K713 and K714 maintain the orientation of the downstream DNA (Nayak et al., 2007). The role of the thumb subdomain in the stability of the elongation complex is also explored. It is theorized that this motif acts such as a sliding clamp once bound to the template DNA and interacts with the growing RNA via positively charged residues (Mukherjee et al., 2002).

The translocation of RNAP along the DNA during RNA synthesis proceeds via the following two steps. In the first step, after the phosphodiester bond formation and release of PPi, the EC is in a “pre-translocated” position. In this state, the RNA still occupies the position in which the incoming NTP should bind to extend the transcript. In the second step, conformational changes that lead to the extension come after the PPi released from the phosphodiester bond interacts with the finger subdomain (open state) and O-helix interacts with incoming NTP (closed state). This step, called the “post-translocated” position, achieves the transfer of the substrate to the insertion site, translocation of RNA:DNA duplex (function of Y639 displacement in T7 RNAP), opening of the downstream DNA (by 1 bp) and closing/reannealing of upstream DNA. These open and closed states drive the translocation of the EC along the DNA (Yin and Steitz, 2002; Temiakov et al., 2004; Steitz, 2009). The template sequence also affects the forward translocation, and it has been reported that the *K*_*m*_ for the elongating NTP is affected by the ease or hindrance to forward translocation (Thomen et al., 2008). Fidelity of this highly processive state can be owed to the base pair interactions the incoming NTP has with the nucleotide on the template strand; these tend to be fast and the right base pairing increases the residence time for interactions. A factor that might increase the residence time for these incoming nucleotides may decrease the fidelity (use of divalent metal ions with stronger coordination than Mg^2+^) (Pezo and Wain-Hobson, 1997).

CMAs relating to template DNA and the RNAP affect the EC’s processivity. As mentioned above, the template DNA sequence regulates nucleotide incorporation speed. The structural stability of the RNAP also impacts the EC; early purification strategies for T7 RNAP after overexpression in bacterial cells resulted in structural damage of the NTD due to nicking between residues K172 to K179 (Muller et al., 1988). Studies of the processivity of this nicked enzyme and RNAP with extensively degraded NTD have shown either highly reduced processivity or complete dissociation in the elongation phase. Structural studies have shown that this nicked region of the RNAP forms a part of the RNA exit channel, and the disruption in the interaction of the growing RNA strand with the exit channel affects the processivity of the EC (Yin and Steitz, 2002; Koh et al., 2018). CPPs related to IVT reactions, such as nucleotide concentration, RNAP concentration (instability from enzyme bumping), co-factor concentration, and reaction temperature, also affect the EC and its processivity. The inherent stability of the EC or the RNAP conformation in the EC may also result in the synthesis of product-related impurities such as dsRNA (Dousis et al., 2023). As mentioned above, the conformational changes in the RNAP during elongation catalyze the RNA polymerization in a sequence-independent manner; the RNAP binds to 3′looped RNA and proceeds with its extension without requiring promoter recognition (Gholamalipour et al., 2018). Indeed, newer T7 RNAP (G47A) mutants showing less dsRNA generation indicate stabilizing IC relative to the EC (Dousis et al., 2023; Rabideau et al., 2019). This hypothesis requires further studies and comparison of run-off vs. terminator sequence-dependent termination.

### 2.6 Transcription termination

The highly processive elongation phase ends once the EC encounters specific terminator sequences or simple dissociation from the lack of downstream DNA (linearized DNA). In hyper-forward translocation, the RNAP is pushed forward by secondary structures (hairpin loop) and a stretch of downstream U residues in the synthesized RNA (Lyakhov et al., 1998; Zhou et al., 2007; You et al., 2023). Sequences with a high degree of base complementarity and base pairing strength drive the formation of these structures in the RNA; this, along with a weaker base pairing of U (in RNA) with A (in template strand), facilitates the opening of the hybrid topological lock (Zhou et al., 2007). The T7 RNAP has a corresponding terminator sequence found in the phage genome; this sequence is inherently weak at termination, with reported efficiency between 60%–80% (Calvopina-Chavez et al., 2022). Sequence-dependent terminators can be divided into structured class I (hairpin-forming) and unstructured class II sequences. The former achieves termination by inducing hyper-translocation (3′OH group of the RNA is removed from the active site, ending the catalytic cycle). At the same time, the latter is theorized to collapse the transcription bubble that leads to DNA unbending and translocation from restraining interaction with upstream DNA (Macdonald et al., 1994). Regardless of the mechanism, the outcome unthreads the RNA from the topological lock with the template strand. Class I terminators for phage polymerases have a more stable stem and longer U-run than similar termination structures for bacterial polymerases. Novel hairpin structure coding terminator sequences with at least 12 internal base pairs and 60% GC content have been proposed to offer better termination efficiencies (Striedner and Wittwer, 2019). Secondary structure stabilization can be estimated by Gibbs-energy (ΔG); higher stability pertains to lower ΔG (Mairhofer et al., 2015). It was shown that structures with stems greater than 9 bp, even after decreasing the ΔG, did not improve termination efficiency (for bacterial terminator sequences).

In contrast to the mechanism of class I terminators, where RNA structure is responsible for termination, class II terminators do not form these structures; this suggests a fundamental difference in the termination mechanism between these two sequences. Class II terminators were originally isolated from the human prepro-parathyroid hormone (PTH) gene (He et al., 1998). Later, similar terminators were found in the concatemer junction (CJ) of bacteriophage DNA, *E.coli* rrn BT1 terminator and cDNA copy of vesicular stomatitis virus (VSV). As it is not the RNA structure that drives termination in a class II sequence, the overall mechanism was more complex to decipher. It was shown that class II terminators must be present as a duplex and that the nontemplate strand was crucial in its functioning; base changes within the PTH terminator were shown to reduce the terminator efficiency. The conserved class II sequence was determined to be the 7 bp ATCTGTT (ATCTGTTTT for T7 RNAP); the upstream sequence, although important, is not absolutely required. A U-run downstream of the main sequence is a standard feature for both class I and II terminators. Moreover, shortening of the four U residues or their substitution prevents termination by class II sequences (He et al., 1998). Class II terminators have been found to be better suited for IVT application, but newer terminator sequences based on the combination of class I and II terminator sequences have proven to be highly efficient in both *in vitro* and *in vivo* applications. These constructs have shown more than 90% termination efficiency (Mairhofer et al., 2015; Striedner and Wittwer, 2019; Calvopina-Chavez et al., 2022).

Run-off transcription using a linearized template DNA is a hallmark of the IVT reaction. This mechanism partly gives the IVT reaction its high turnover. The enzyme elongates on the template DNA until the end of the template DNA, e.g., obtained by plasmid linearization, and the T7 RNAP slides off. The forward hyper translocation of the RNAP is favoured in run-off transcription as the regulation from melting the downstream DNA is absent at the end of the template, which, combined with reannealing of the template and nontemplate strand, results in the collapse of the transcription bubble and dissociation of the RNA:DNA hybrid. The productivity (from high turnover) in a run-off transcription is much higher compared to one in which internal dissociation terminates the elongation phase. It presents its own challenges as the EC becomes very unstable at the end of the template and adds nucleotides to the 3′end of the intended transcript. 3′heterogeneity is a major problem and leads to “nontemplated” nucleotide addition (N+ *x* additions; here, N is the intended transcript length). Recent results have shown that these additions depend on the template-dependent (cis-primed extension of 3’looped back RNA) (Gholamalipour et al., 2018).

The termination phase of transcription by bacteriophage-derived RNAPs might be the least studied in optimizing the IVT reaction. As mentioned above, several product-related byproducts are generated due to improper termination of the processive elongation phase; the characteristics that make IVT productive could potentially be responsible for such an outcome. CMA improvements related to template DNA that facilitate sequence-dependent termination, linearization (of plasmid DNA) without 3’overhangs (Rong et al., 1998b), and transcript sequence optimization for reducing complementarity to prevent 3’loop back could be one area for overall process optimization. CMAs related to the RNAP have also been extensively explored. Mutant RNAPs that give much better 3’homogeneity compared to wild-type T7 (Wu et al., 2021; Dousis et al., 2023) RNAP are documented. Thermostable mutant RNAPs (Ong et al., 2022; Roy and Robb, 2022) that take advantage of higher process temperature to disrupt loopback formations in the synthesized RNA and alternative bacteriophage RNAPs, such as those derived from cyanophage Syn5 (Zhu et al., 2013), have been reported to give much better 3’homogeneity compared to the current standard wild-type T7 RNAP. Any approach that can revert the RNAP to IC without proceeding to an RNA-dependent RNA polymerization after termination and dissociation could help lower the generation of dsRNA byproducts. CPPs associated with IVT that directly influence termination need to be assessed; this includes process temperature, concentration of RNAP, concentration of non-canonical nucleotides, presence of ions such as Mg^2+^ or arginine (in case of class II terminators) and the presence of chaotropic agents that disrupt secondary structure (in case of class I terminators).

## 3 Mutant T7 RNAPs and alternatives based on structure-function relationship

An insight into the structure-function relationship was given in the previous section; it touched upon some important residues within the T7 RNAP that were the key enablers of the transcription phase-associated functions. Since there is considerable homology between the standard bacteriophage RNAPs used for manufacturing applications, the insights from one can be extrapolated to a certain degree onto the others (Cermakian et al., 1997). As T7 RNAP is the most characterized, this section will focus on it; crystal structures of T7 enzyme during the various phases of a transcription reaction are well documented (Sousa et al., 1989; Sousa et al., 1993; Sousa, 1997; Cheetham and Steitz, 1999; Yin and Steitz, 2002). The same structures have played a crucial role in understanding the underlying mechanisms of the transcription reaction. The T7 RNAP can be broadly divided into the NTD and CTD. NTD extends from residues 1 to 312, while CTD extends from 313 to 883 (Sousa and Mukherjee, 2003; Sousa, 2013). The CTD is also synonymous with the polymerase domain; this can be further subdivided into the fingers (residues 541 to 737 and 771–778), palm (residues 411 to 448, 532 to 540, 788–838) and thumb (residues 330–410) subdomains (refer Figure 2). Apart from the CTD polymerase domain, which resembles the shape of a cupped right hand, there are accessory modules that are present in the T7 RNAP; these include the NTD, the promoter recognition loop (739–770), C-terminal loop (residues 820–883) and extra 4-helix bundle (residues 449–531). These modules enable promoter recognition, duplex opening, RNA binding and displacement and transcription regulation (Sousa and Mukherjee, 2003). An extensive list of mutant T7 RNAPs (from patent documents and research articles) based on proposed improvements to structure-function relationships is given in Table 1. Although these mutant T7 RNAPs provide extended capabilities for non-canonical substrate utilization and reduction in product-related impurities, they require extensive characterization. Properties such as fidelity requires further investigation before they can be used in large-scale manufacturing of mRNA-based products.

### 3.1 Amino-terminal domain (NTD)

The NTD has four motifs that perform crucial roles; these include: i) promoter binding domain (PBD), a six-helix subdomain (residues 72–150 and 191–267) that interacts with the AT-rich upstream region of the promoter; ii) H domain, a two-helix subdomain (residues 151–190) that forms part of the RNA exit channel; iii) C-helix (residues 28–71), responsible for active site enlargement and iv) C-linker (residues 251–296), facilitates transition from IC to EC by providing structural flexibility (Sousa and Mukherjee, 2003; Martin and Ramirez-Tapia, 2015; Dousis et al., 2023). The NTD undergoes drastic conformational changes during the transition from IC to EC, facilitating the high processivity of the T7 RNAP seen during the elongation phase. Positively charged residues (93–101) of the PBD interact with the minor grove of the promoter (−17 to −13 bp) and the intercalating loop (residues 232–242) with V237 facilitates the opening of the duplex by stacking on the −5 bp and stabilizing the upstream edge of the open complex (Brieba and Sousa, 2001; Yin and Steitz, 2002; Tang et al., 2008; Tang et al., 2009). The H subdomain shows significant movement during the transition from IC to EC. It moves towards the active site and becomes part of the RNA exit channel and the other accessory module (promoter recognition loop) (Tang et al., 2009). Studies on nicked T7 RNAP (in the H subdomain) have shown decreased activity in the elongation phase, and this could be attributed to the disruption in the RNA exit channel, which is partly formed by the H subdomain (Muller et al., 1988). C-linker and C-helix also have an active role in the transition of IC to EC; the former facilitates the enlargement of the active site during the initial transcription, especially for the accommodation of the template DNA from scrunching. Conversely, C-linker helps with the structural flexibility of the NTD and facilitates its movement (Sousa and Mukherjee, 2003; Dousis et al., 2023). The NTD of T7 RNAP has been subjected to various mutations to improve the industrial application. As is evident from Table 1, thermostability-conferring mutations are the most common when it comes to NTD mutations. The mutations to C-linker and C-helix have also been employed to reduce the synthesis of abortive transcripts or reduce the RNA-dependent RNA polymerase activity of the enzyme. It has been speculated that stabilization of EC to IC after termination/dissociation might be the key to reducing product-related impurities such as dsRNA and 3′heterogeneous products. Regardless, NTD modifications can reduce product-related impurities passively by relying on increased process temperatures or actively by reducing cryptic promoter-independent RNA-dependent RNA polymerase activity.

### 3.2 Carboxyl-terminal domain (CTD)

The polymerase domain is the larger entity within the T7 RNAP and structurally looks like a cupped right hand. It is responsible for the catalysis of NTPs to the RNA polymer. The subdomains are named finger, palm, and thumb, respectively, each responsible for a particular function during the transcription.

#### 3.2.1 Fingers subdomain

The finger subdomain interacts with the incoming NTP and the template strand downstream of the templating base. The O-helix, part of a five-helix motif within the finger subdomain, interacts with the incoming NTPs using K631 and R627 (positive residues interact with the triphosphate group of the incoming NTP) (Temiakov et al., 2004; Steitz, 2009). Y639 at the tip of the O-helix plays the role of nucleotide insertion to the active site, and the same residue is responsible for substrate discrimination (ribose vs. deoxyribose) based on Mg^2+^ mediated interaction with 2′OH group on the ribose sugar (Brieba et al., 2002; Temiakov et al., 2004). It should be noted that H784 and G542 also play a role in substrate discrimination between ribonucleotides and deoxyribonucleotides (Temiakov et al., 2004; Steitz, 2009). G542 provides less steric clash with 2′OH group, while H784 makes hydrogen bonds with 2′OH of the ribonucleotide. These residues are highly conserved in all phage-like DNA-dependent RNA polymerases. In DNA polymerases, G542 will be replaced with a bulkier residue such as glutamic acid (Cheetham and Steitz, 1999). The movement of the finger domain facilitates the translocation of the RNAP on the template DNA; Y639 pushes the RNA:DNA hybrid, while F644 coordinates the downstream template motion (Da et al., 2017). Moreover, as this domain is responsible for substrate binding and incorporation in the active site, it regulates the fidelity of the RNAP. Multiple modifications have been done to the finger subdomain as it is relevant in substrate incorporation, fidelity, and to an extent the processivity of the RNAP. The earliest T7 mutants were designed to incorporate non-canonical NTPs to the RNA polymer, including 2′-fluoro, 2′-O-methyl base incorporations (refer Table 1). Recently, the incorporation of modified NTPs for better *in vivo* activity has gained traction, especially for specialized therapeutics (Zhu et al., 2022). In such a scenario, mutant T7 RNAPs capable of such synthesis at scale will become more relevant. Increasing the specificity for cap analogs used in co-transcriptional capping is also another area where mutant T7 RNAPs can be specifically useful. Proprietary co-transcriptional cap analogs are the main raw material cost contributors; moreover, their poor utilization further increases the associated manufacturing costs. T7 mutants capable of higher specificity for these cap analogs will reduce the manufacturing cost due to less raw material requirement (Miller et al., 2022). Apart from this, modifications to the finger subdomain have also provided better thermostability to the enzyme. Another interesting modification (C723S) in the finger subdomain has provided better stability of the enzyme, reduced the formation of homodimers due to the disulfide bridge between cysteine residues, and improved the overall enzymatic activity (Greif et al., 2017).

#### 3.2.2 Palm subdomain

The palm subdomain houses the active site of the RNAP, and the highly conserved aspartate residues at D537 and D812 facilitate the formation of phosphodiester bonds (Woody et al., 1996). These residues coordinate with 2 Mg^2+^ ions that stabilize the pentacoordinate phosphorous intermediate and facilitate nucleophilic attack of the 3′-OH on the RNA terminal nucleotide by the α phosphate of the substrate NTP (two-metal ion mechanism) (Yang et al., 2006). PPi released as a result of this reaction coordinate with the finger subdomain to drive the translocation of the polymers (Steitz, 2009). Although T7 RNAP is a highly helical enzyme, the RNA and DNA facing side of the palm subdomain is composed of *β* sheets (Cheetham and Steitz, 1999). The residues responsible for catalysis are highly conserved and any changes here are detrimental to the activity of the RNAP; however, T7 mutants with thermostable properties are engineered by changes in the palm subdomain (most of the time in combination with changes in the finger subdomain). The palm subdomain also remains conserved across various RNA and DNA polymerases.

#### 3.2.3 Thumb subdomain

The thumb subdomain is vital for the stabilization of the EC, it wraps around the DNA to form a clamp which would prevent dissociation of the complex during translocation on the DNA. The growing RNA:DNA hybrid interacts with positively charged residues on the thumb subdomain and facilitates its stabilization (Durniak et al., 2008). Mutations in the thumb subdomain to neutral residues increase the dissociation of RNA. Like mutants derived for the palm subdomains, thumb subdomain mutations result in thermostable properties.

#### 3.2.4 Accessory modules

Apart from changes in the main subdomains, mutations in the accessory modules (apart from NTD) are quite rare; in our assessment, we did not find any mutant with changes to the promoter recognition loop (anti-parallel *β* ribbon). Changes to the C terminal loop are also rare, as it was previously reported that any changes in this motif are detrimental to the enzyme’s activity. F882 provides a binding site to the elongating NTP, and any changes to the F880, A881, F882 and F883 affect the catalytic efficiency (Gardner et al., 1997). Insertional mutants with smaller residues after F883 were found to be still active and based on this, a G884 mutant was engineered that showed better 3′homogeneity of the transcripts compared to wild-type T7 RNAP (Dousis et al., 2023; Rabideau et al., 2019). The extra 4-helix bundle is yet to be characterized for its function; so far, we have found only one mutant that confers thermostability to the enzyme from changes in this accessory module.

### 3.3 Phylogeny and alternatives to T7 RNAP

**FIGURE 6:**
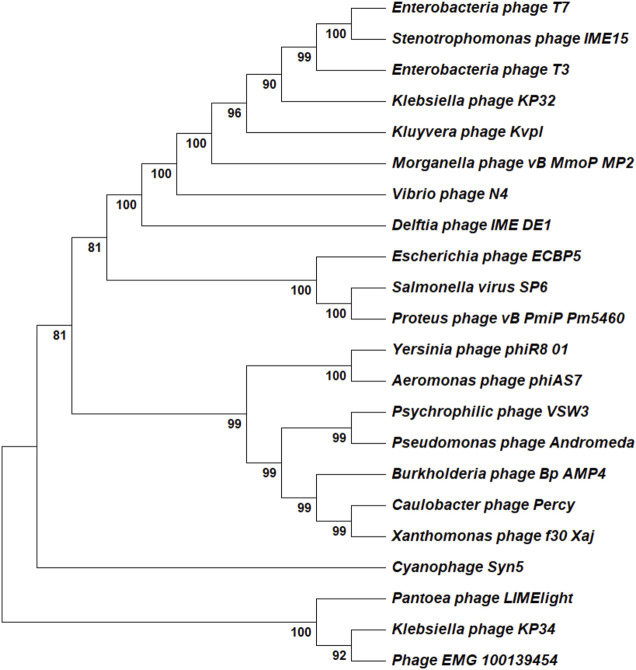
Phylogenetic analysis of T7 RNAP alternatives. All the reported alternatives are single-subunit RNAPs and the analysis was done using a simple neighbor-joining tree. The analyzed RNAPs belong to the *Autographiviridae* family.

The T7 RNAP shows structural similarity with other RNAPs and DNAPs as well as limited similarity with the polymerase domain of reverse transcriptase. T7 RNAP is a member of the single-subunit RNAPs, which includes other bacteriophage RNAPs, nuclear gene-coded mitochondrial RNAPs and chloroplast RNAPs (Cermakian et al., 1997). The accessory modules described in the previous section provide the most significant variation between these related enzymes. Similarities with DNAPs have prompted using mutants capable of incorporating rNTPs as a possible alternative for bacteriophage-derived RNAPs. Mostly, these alternatives are isolated from extremophiles, which gives the added advantage of thermostability (Wang et al., 2017). Structural similarity between T7 RNAP and Pol I class of DNAPs suggests they come from a common ancestor. However, the cellular multi-subunit RNAPs lie outside this superfamily. Based on the phylogenetic analysis, T7 RNAP alternatives could be used to meet specific industrial applications. The product-related impurities generated by the wild-type T7 RNAP could be avoided with alternatives derived from other bacteriophages or extremophile bacteria. A few of the newly characterized alternatives include RNAPs derived from *Klebsiella* phage (KP34), Cyanophage (Syn5), and *Pseudomonas* phage (VSW-3) (Zhu et al., 2013; Lu et al., 2019; Xia et al., 2022). The RNAPs from the alternatives provide several advantages over the wild-type T7 RNAP, including higher processivity (useful for the synthesis of longer RNA constructs such as self-amplifying mRNA), reduced 3′heterogeneity, reduced dsRNA generation and improved incorporation of modified NTPs. We have summarized a list derived from patent applications and research articles of potential T7 RNAP alternatives that may have industrial usefulness in Table 2 and showed the phylogenetic relationship between these alternatives using a neighbor-joining phylogenetic tree in Figure 6. Apart from using mutant T7 RNAP and alternative bacteriophage-derived RNAPs, a new solution for mRNA-based products comes from fusion proteins; here, T7 or related RNAPs are fused with another enzyme capable of activities such as 5′capping (Chan et al., 2023). Such a solution for producing capped mRNA can have significant manufacturing cost benefits. Currently, enzymatic capping is resource intensive as mRNA produced from the IVT process needs to be purified before the capping reaction is done using another enzymatic reaction (usually done with vaccinia or faustovirus capping enzymes). Fused enzymes with polymerase and capping activity can reduce/circumvent the dependence on proprietary capping reagents and reduce the raw material costs and operating costs, as fused enzymes grant a one-pot synthesis and capping reaction.

### 3.4 Methods for RNAP engineering

The review has highlighted many mutants that are useful for specific industrial applications; therefore, another question that arises is how these mutants are screened and engineered; this could be broadly divided into directed evolution and rational design. While the former does not require information about structure-function properties, the latter heavily relies on the sequence and structure-function relationship and aims to introduce changes via methods such as site-directed mutagenesis (Reetz et al., 2023). Directed evolution introduces random mutations via methods like error-prone PCR or DNA shuffling; continuous directed evolution and phage-assisted continuous evolution (PACE) are newer iterations of this method (Packer and Liu, 2015; Miller et al., 2020). The mutants highlighted in the table were engineered via rational design and directed evolution methods such as PACE. The cost and time associated with screening and engineering of useful mutant enzymes can be substantial; however, strategies such as adaptive machine learning are reducing this burden and helping screen mutants with fewer evaluations (Hie and Yang, 2022). Apart from screening of mutants, improvements in phylogenetic analysis have also helped with the identification of potential alternatives for commonly used T7 RNAP; regardless of whether screening for mutants or T7 alternatives, computational methods assist with high throughput screening while reducing overall resource utilization and associated costs.

## 4 QbD and economics of mRNA manufacturing

As mRNA-based products are gaining traction and newer varieties like self-amplifying and circular mRNA are being considered for use in prophylactic and therapeutic applications, optimizing the process that enables manufacturing these drug substances becomes crucial. Implementing the quality by design (QbD) paradigm for mRNA manufacturing is being discussed extensively, and this approach complements the ‘platformability’ inherent to mRNA manufacturing (Daniel et al., 2022; Whitley et al., 2022; Nair et al., 2024). Throughout this review, we focused on a central component of this manufacturing process, the enzyme that enables the production of the drug substance. From a QbD perspective, the CMAs and CPPs that affect the CQAs and KPIs do so by influencing the functionality of the RNAP. Moreover, it can also be seen that different CMAs and CPPs affect the CQAs by influencing the enzyme in its various phases during the IVT reaction. As noted, the CPPs, such as ionic strength, have more relevance at the initiation than at the transcription reaction’s elongation or termination phase. Substrate concentration, co-factor concentration, type of buffering agent, type of template DNA, sequence of template DNA, pH, reaction time and temperature are some of the few CMAs and CPPs that affect this process. Mapping the relationship between CMAs/CPPs and CQAs is essential in the QbD paradigm. This could be improved further by understanding the fundamental mechanisms involved in the process. Traceability and reagent quality used in manufacturing are other crucial aspects to consider when establishing a stable and capable process; often, this is achieved with good manufacturing practice (GMP) compliance. This approach also facilitates troubleshooting in case of quality failures and maintains consistency in the quality of the final product. GMP-graded reagents increase the overall production costs, but adapting them at the early stage of development can aid in entering clinical trials and reaching regulatory approvals faster. Importantly, GMP-graded reagents do not always have higher-quality than reagents sold for research and development purposes. However, GMP-grade reagents are: 1) produced using production processes validated to ensure consistency and reproducibility, 2) undergo stringent quality control and testing, 3) include thorough documentation, offering traceability of raw materials, batch records, testing results, and certificates of analyses. RNAPs used in mRNA manufacturing are manufactured and formulated like any other reagents; therefore, GMP compliance that ensures consistent functioning should be followed. Certificates of analysis with enzyme activity and purity and a list of ancillary materials should be provided. Moreover, these criteria may change depending on any additional modifications to the RNAP, including changes to the expression host (e.g., *Escherichia coli*) and plasmid vector containing the RNAP gene sequence.

The development of computational models for better process monitoring and control is the next step in process optimization/improvement; these models can either be data-driven or mechanistic models (based on reaction kinetic or mass balance) (Hengelbrock et al., 2023). Data-driven models are black boxes that solely rely on process data and are robust within the model input-output variable space (capable of mostly interpolation). On the other hand, mechanistic models are robust and can be capable of extrapolation, but they are relatively more difficult to develop and computationally complex. Hybrid models that combine the advantages of both these models could be an ideal solution, but this will require better process characterization (Nair et al., 2024). Looking at the IVT reaction based on the influence of CMAs/CPPs on the RNAP and the CQA of the synthesized process output will undoubtedly benefit better process and quality control. Furthermore, the transition to bioprocess 4.0 will also require adopting digital tools such as soft sensors and digital twins that rely on process data and a robust computational model derived from a well-characterized process (Isoko et al., 2024).

**FIGURE 7:**
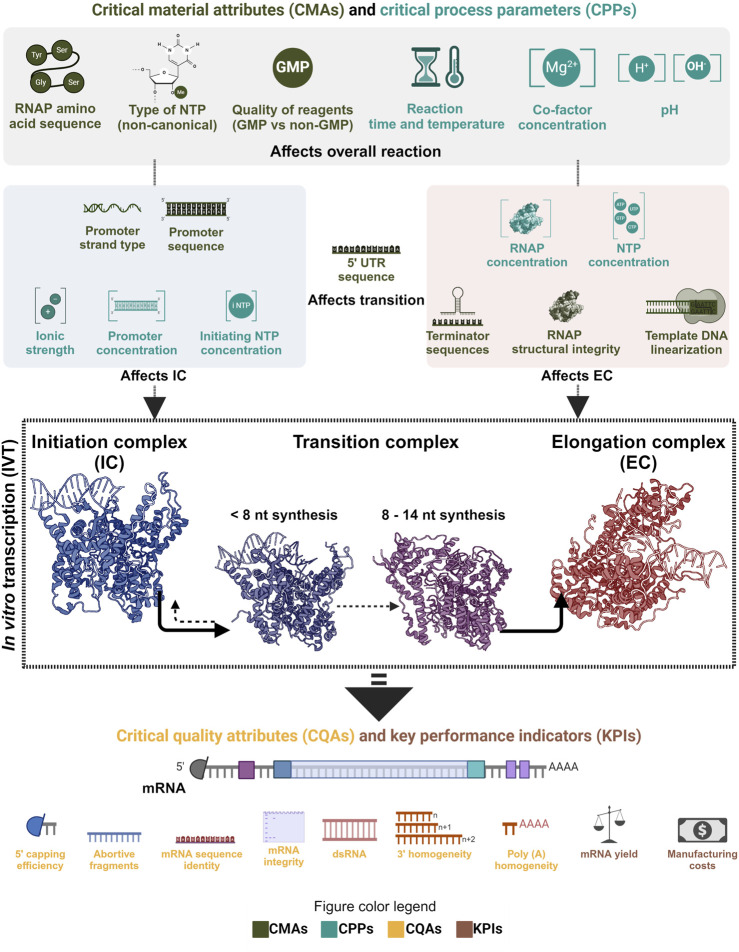
Effect of CPPs and CMAs on the T7 RNAP during the IVT reaction. CPPs and CMAs that affect the overall reaction are grouped in the grey box, the ones that affect the IC are grouped in the blue box and the ones that affect the EC are grouped in the red box. QbD implementation based on the insights gained from these interactions would serve to optimize the CQAs and KPIs of the final product and manufacturing process, respectively. Initiation complex with synthesis up to 3 nucleotides is depicted by PDB-1QLN. The transition complexes with the synthesis of RNA up to 8-nt is depicted by PDB-3E3J and the synthesis of 8–14-nt is depicted by PDB-1MSW. The elongation complex is depicted by PDB-1S76.

In the end, the efficiency of the manufacturing process determines the economic viability of the drug product and if the manufacturing costs associated with a drug modality are high, its wider adoption is severely hindered. As mRNA-based vaccines have proven their effectiveness, more and more products are being designed with this technology for both prophylactic and therapeutic applications. Currently, mRNA manufacturing is expensive, with raw material costs dominating the operating expenditure; most of this can be attributed to the use of proprietary reagents such as cap analogs used in the co-transcriptional capping of mRNA. As mentioned in the previous sections, strategies that can reduce the amount of these reagents or circumvent them completely can drastically reduce production costs (Kis et al., 2020b). Moreover, product-related impurities, such as immunogenic dsRNA, need to be separated from the drug substance before it can be formulated into the drug product. Downstream purification processes are another major cost contributor to the overall manufacturing process (Kis et al., 2020b). If the generation of unwanted byproducts can be reduced, it can have huge implications for the costs associated with downstream processes. Some of the product-related impurities are very challenging to remove even with advanced purification strategies; this reduces the effective yield from the IVT process and the downstream purification processes. Indeed, modifications to the T7 RNAP, as previously described, aim to reduce the overall manufacturing costs. The RNAP is also a significant contributor to the cost of raw materials, and therefore, strategies to reduce this should be seriously considered. Simple strategies can be effective, such as optimizing RNAP concentration in the IVT reaction to avoid reagent excess. More complex strategies could involve a change in the mode of operation for the IVT reaction; recently, a shift from batch mode of operation to fed-batch was suggested to produce large quantities of mRNA all the while using relatively less amount of RNAP (Elich et al., 2020; Skok et al., 2022a; Pregeljc et al., 2023; Boman et al., 2024; Guo et al., 2024). Enzyme reuse/recycling could be another method for cost reduction. Immobilized T7 RNAP is reported to be reused in multiple cycles of IVT reaction. Moreover, some modifications with immobilized T7 RNAP have also been suggested to reduce the synthesis of product-related impurities, thus reducing downstream process-related costs. If we look at the IVT reaction closely, we can see that optimizing the catalyst can be the solution to making mRNA manufacturing more cost-competitive. There have been multiple studies done to optimize the large-scale IVT reaction, but these have yet to include a fundamental understanding of how RNAPs influence the final quality of the product. As summarized, there are CMAs and CPPs that do not directly influence the final product; they influence the RNAP that synthesizes the mRNA and improving its functionality may hold the key to the success of this drug substance. Figure 7 summarizes the effect of CPPs and CMAs on the T7 RNAP during the IVT reaction as discussed.

## 5 Conclusion

In this review, we assessed the role of bacteriophage-derived RNAPs in the IVT reaction from a QbD perspective, as these enzymes are at the center of the IVT reaction that has enabled the synthesis of a drug substance class that has gained significant traction ever since the success of mRNA vaccines during the SARS CoV2 pandemic. Decades of fundamental research have enabled extensive characterization of the RNAPs used for large-scale mRNA synthesis. Combining this wealth of knowledge with the QbD approach can improve the manufacturing process and make it highly efficient. We provided a historical background to the discovery and initial characterization of the most used bacteriophage-derived RNAPs and used T7 RNAP to represent industrially applicable single-subunit RNAPs to explain the various phases of the transcription reaction. The influence of CMAs and CPPs involved with the IVT reaction and their direct influence on the RNAP was also considered; this should help implement the QbD approach from a mechanistic perspective. Moreover, we tried to combine the structure-function relationship and industrial applicability of the T7 RNAP and several of its mutants. Alternatives to T7 RNAP were also discussed, although it should be noted that industrial use of any of the less characterized alternatives will likely have significant regulatory hurdles. An advantage of wild-type T7 RNAP is its extensive characterization, which lowers risks and increases the likelihood of faster regulatory approvals. We concluded the review by emphasizing the influence of RNAP on the economics of the mRNA manufacturing process. Considerable improvements to the production costs can be achieved by the use of RNAPs that reduce raw material utilization (pertaining to cap analogs) or circumvent the use of proprietary reagents altogether (single-pot synthesis and enzymatic capping). The reuse/recycling of RNAPs can also reduce manufacturing costs and improve the overall sustainability of the manufacturing process.
